# CT-based radiomics models for predicting the prognosis of children with mycoplasma pneumonia

**DOI:** 10.3389/fcimb.2026.1737753

**Published:** 2026-03-24

**Authors:** Tianda Wang, Huan Meng, LiYong Zhuo, Lili Zang, Jingjing Cui, Jianing Wang, Xiaoping Yin

**Affiliations:** 1 Department of Radiology, Hebei Key Laboratory of Precise Imaging of Inflammation Related Tumors, The Affiliated Hospital of Hebei University, Baoding, China; 2 Department of Radiology, Baoding Children’s Hospital, Baoding, China; 3 Department of Research and Development, United Imaging Intelligence, Beijing, China

**Keywords:** CT, mycoplasma, pneumonia, prognosis, radiomics

## Abstract

**Background:**

In recent years, the incidence of mycoplasma pneumonia (MP) in children has gradually increased; however, to date, few studies have assessed its prognosis in children. Hence, the present study aimed to develop a prognostic model for children with MP by using clinical data and radiomics features extracted from chest computed tomography (CT) images.

**Methods:**

A total of 356 children with MP from two hospitals were enrolled in the study. These patients were randomly assigned to a training set (n = 206), a test set (n = 52), and a validation set (n = 98). Clinical data, including patients’ history and demographics, laboratory test results, and CT imaging features, were collected. Radiomics features of the infected lesions were extracted from chest CT images. Univariate analysis was performed to determine the most predictive features with significant differences (P < 0.05) among all imaging and clinical data. Least absolute shrinkage and selection operator was used to select radiomics features and estimate the prediction performance of the clinical factors model and the radiomics model for prognosis.

**Results:**

In the training set, the clinical factors (creatine kinase-MB, lactate dehydrogenase, C-reactive protein, bilateral lobe pneumonia, number of lobes, and lesion volume) showed a significant prognostic value (all P < 0.05) and were selected to construct the clinical model. The area under the curve (AUC) values of the clinical model for the training, validation, and test sets were 0.767, 0.731, and 0.685, respectively. Twenty one radiomics features were selected. The AUC values of the radiomics model for the training, validation, and test sets were 0.829, 0.775, and 0.701, respectively. Decision curve analysis showed that the radiomics model has potential clinical application value for predicting the prognosis of children with MP. The clinical and radiomics model showed the best prediction performance, with AUC values of 0.842, 0.825, and 0.748 for the training, validation, and test sets, respectively.

**Conclusion:**

The radiomics model based on CT imaging features showed potential as a quantitative tool to predict the prognosis of children with MP. The addition of radiomics features to the clinical factors improved the diagnostic efficiency of the clinical and radiomics model. Thus, the combination of clinical data and radiomic features effectively predicted the prognostic outcome of children with MP.

## 1 Introduction

Mycoplasmal pneumonia (MP) is a contagious respiratory disease usually caused by Mycoplasma pneumoniae; it is more commonly diagnosed in children and has shown an increasing incidence in recent years. Clinical manifestations of MP mainly include symptoms such as cough, fever, sputum production, shortness of breath, and chest pain (Wu et al., 2023). During the early phase of MP infection, the diagnosis and assessment of the disease could be complicated by a diverse range of symptoms and radiological presentations, coupled with variations in disease severity upon onset (Luo et al., 2023). Additionally, because of individual differences in immune response and varying degrees of infection, the prognosis of MP shows significant variability. Therefore, early noninvasive prediction of the prognosis of MP patients is crucial for appropriate clinical diagnosis and treatment.

Chest computed tomography (CT) is a critical modality to diagnose, monitor disease progression, and evaluate curative effects in clinical settings. Shir et al (Shiri et al., 2021). found that in patients with COVID-19, CT could predict which patients might progress to severe disease; this making it possible to implement early intervention, which partly reduced the incidence of severe COVID-19 and improved patient prognosis. The clinical value of CT imaging relies mainly on the early detection of lung infections and high accuracy in quantifying disease progression and severity. However, chest CT has limited application in identifying specific respiratory disease-causing viruses. Moreover, the role of CT in the diagnosis and conditional evaluation of MP also remains controversial.

As an emerging interdisciplinary and cross-disciplinary technology, radiomics can extract highly informative quantitative features from radiographic images. The extracted radiomics features provide complementary insights to radiologists for conducting qualitative analyses (Shi et al., 2022). By using radiomics features, data-driven models can be trained to predict clinical outcomes. Current research has shown an improvement in the diagnostic capability of doctors with the support of quantitative radiomics features. The key advantage of radiomics features is their intrinsic interpretability, i.e., the meaning conveyed by each feature is well-known. Several studies have used deep or machine learning (ML) algorithms for predicting COVID-19 prognosis; the results indicated that these approaches could serve as alternative methods for prognosis prediction or evaluation of patient response to therapy (Ferreira Junior et al., 2021; Shiri et al., 2022; Sun et al., 2023). However, the role of radiomics features in predicting the prognosis of children with MP remains unclear.

In the present study, we developed radiomics and clinical and radiomics models based on the features extracted from CT images of children with MP. We integrated the radiomics model with clinical characteristics and conventional CT features for the noninvasive prediction of the prognosis of children with MP, offering valuable insights for the clinical diagnosis and treatment of MP in children.

## 2 Materials and methods

### 2.1 Patients

This retrospective two-center study included children with Mycoplasma pneumoniae pneumonia (MP) who underwent chest CT examination at the Affiliated Hospital of Hebei University (Center A) and Baoding Children’s Hospital (Center B). All patients were enrolled according to the same inclusion and exclusion criteria. Clinical, laboratory, and imaging data were retrospectively collected from the electronic medical record systems of the two institutions.

The inclusion criteria were as follows (1): age ≤ 14 years (2), diagnosis of community-acquired pneumonia (3), blood MP-immunoglobulin M (IgM) titer ≥ 1:160 or a 4-fold increase in IgM titer in acute and convalescent serum specimens; and (4) confirmation of M. pneumoniae in bronchoalveolar lavage fluid (BALF) by PCR. The exclusion criteria were as follows (1): presence of underlying diseases, such as hematological diseases, cardiovascular diseases, bronchial asthma, and immunodeficiency disorders (2); lack of clinical data (3); co-infection with pulmonary tuberculosis or pulmonary abscess; and (Sun et al., 2023) poor image quality.

### 2.2 Follow-up

The patients were assigned to favorable and poor prognosis groups according to the symptoms and chest CT examination results. Favorable prognosis was defined based on (1) elimination of or relief from clinical symptoms and signs (2), normal findings in chest CT examination or resolution of most foci, and (3) restoration of serological parameters to normal levels. Poor prognosis was defined as (1) discreet alleviation or aggravation of the clinical symptoms and signs and (2) minor changes and even deterioration in the findings of chest CT examination and laboratory indices (Li et al., 2020).

### 2.3 Clinical factors

Data collected from hospital electronic medical records included demographic characteristics (age and sex), fever course, extrapulmonary complications, and laboratory tests white blood cell (WBC) count; platelet (PLT) count; neutrophil percentage; and levels of C-reactive protein (CRP), procalcitonin (PCT), lactate dehydrogenase (LDH), D-dimer, activated partial thromboplastin time (APTT),Biotin-Avidin—System(BAS),eosinophil count(EOS),Fibrinogen(FIB).

### 2.4 Image acquisition and analysis of imaging results

Philips Brilliance 256-row spiral CT machine, GE Discovery HD750 CT scanner, and United Imaging uCT550 spiral CT scanner were used for imaging. The patient was placed in the supine position with both hands raised above the head. The scanning range was from the thoracic inlet to the diaphragm level. Scanning in the deep breath hold position was performed after deep inspiration. The scanning parameters were as follows: tube voltage, 120 kV; tube current, automatic milliamp technology; pitch, 0.900, 0.984, and 1.175; rotation time, 0.5 s, 0.6 s, and 0.6 s; matrix, 512 × 512; layer thickness, 5 mm; interlayer spacing, 5 mm; and field of view, 40 cm × 40 cm. Axial reconstruction of the lung window and mediastinal window was performed using the following parameters: window width, 1,500 and 350 HU, respectively; window level, 600 and 40 HU, respectively.

CT images were independently reviewed by two radiologists with rich experience in chest imaging diagnosis. For any disagreements, the two radiologists reached a consensus through consultation. The following CT characteristics of each patient were recorded: lobar atelectasis, halo sign, cavitation, pleural effusion, abnormalities found in bronchoscopy (air bronchogram sign or bronchial wall thickening), and number of lobes involved.

### 2.5 Extraction of radiomics features and development of the models

**Figure 1:**
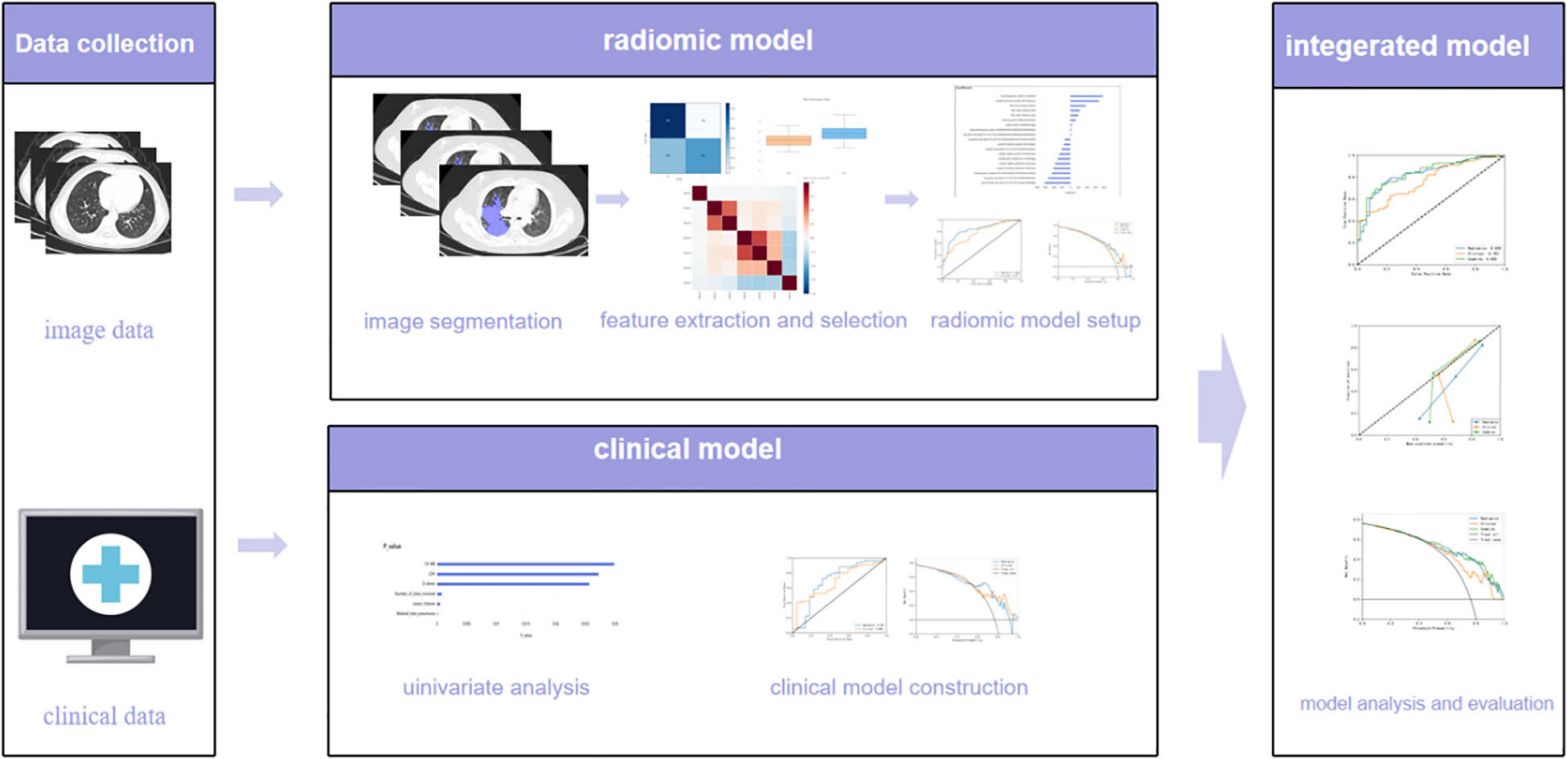
The workflow of different model.

The CT images were standardized before feature selection and model development. The images were resampled to 1×1×1 mm3 voxels by using the B-spline interpolation method. The features were normalized by the Z-score method, in conjunction with lung tissue-specific Hounsfield Unit (HU) window settings (window width, 1500 HU; window level, −600 HU). In addition, N4 bias field correction was applied to reduce scanner-related intensity inhomogeneity. Auto-segmentation, extraction of radiomics features, feature selection, and ML model development were performed using the uAI Research Portal V1.1 (Shanghai United Imaging Intelligence Co., Ltd.) (Cheng et al., 2020; Gu et al., 2021; Shan et al., 2021; Wu et al., 2023). And the region of interest (ROI) was semi-automatically segmented using uAI Research Portal V1.1 and then manually corrected by two independent radiologists with 10 and 12 years of experience in thoracic imaging diagnosis, respectively, to ensure accurate delineation of subtle lesion boundaries. Radiomics features were automatically extracted from regions of interest (ROIs) by using Pyradiomics v3.0, an open-source Python package (Griethuysen et al., 2017). Finally, 2,264 features were extracted from each ROI. Pearson’s correlation coefficient was used to select features with a significant difference between features and labels (P < 0.05). Subsequently, the least absolute shrinkage and selection operator (LASSO) algorithm was utilized for selecting better feature subsets through compression estimation and regularization. **Figure 1** shows the workflow of our study.

Univariate analysis was performed to select CT imaging signs and clinical features showing a significant difference (P < 0.05). Radscore was calculated by assigning weights to the features according to the coefficients obtained by LASSO. Subsequently, multivariate logistic regression analysis was conducted to construct radiomic models based on the features selected. Finally, a combined clinical and radiomics model was constructed using Radscore, CT imaging signs, and the selected clinical features. Grid search with 10-fold cross-validation was used to select the optimal parameters for the models. To further mitigate the risk of overfitting due to the relatively high number of selected radiomics features (n=21) compared to the sample size, we performed additional internal validation using bootstrapping with 1000 iterations on the training set. This approach resampled the training data with replacement to generate bootstrap samples, from which 95% confidence intervals (CIs) for performance metrics (including AUC) were derived. Finally, a receiver operating characteristic (ROC) curve, area under the curve (AUC), sensitivity, specificity, and F1-score for the test set were used to evaluate the performance of these models.

### 2.6 Statistical analysis

Continuous variables with normal distribution were compared by t-test and expressed as ( $\bar{\text{x}}\pm \text{s}$), and those with non-normal distribution were compared by Mann-Whitney test and expressed as median with interquartile range. Between-group comparison of the clinical factors was conducted using the chi-square test or Fisher’s exact test for categorical variables and using Mann-Whitney U test for continuous variables. Pearson’s correlation analysis was conducted to determine the correlations of clinical and CT features with different prognosis. A ROC curve was plotted to confirm the relevance of the serological parameters for evaluating the prognosis of children with MP. Statistical analyses were performed for validation and training cohorts. Wilcoxon rank-sum test was used to assess the Radscore between the two prognosis groups. Sensitivity, specificity, accuracy, and AUC values were calculated to assess model performance. Statistical significance was considered at P < 0.05. All statistical analyses were performed and figures were generated using R software (version 3.0.1; http://www.r-project.org) (Wang et al., 2021).

## 3 Results

### 3.1 Clinical information and clinical model

**Figure 2:**
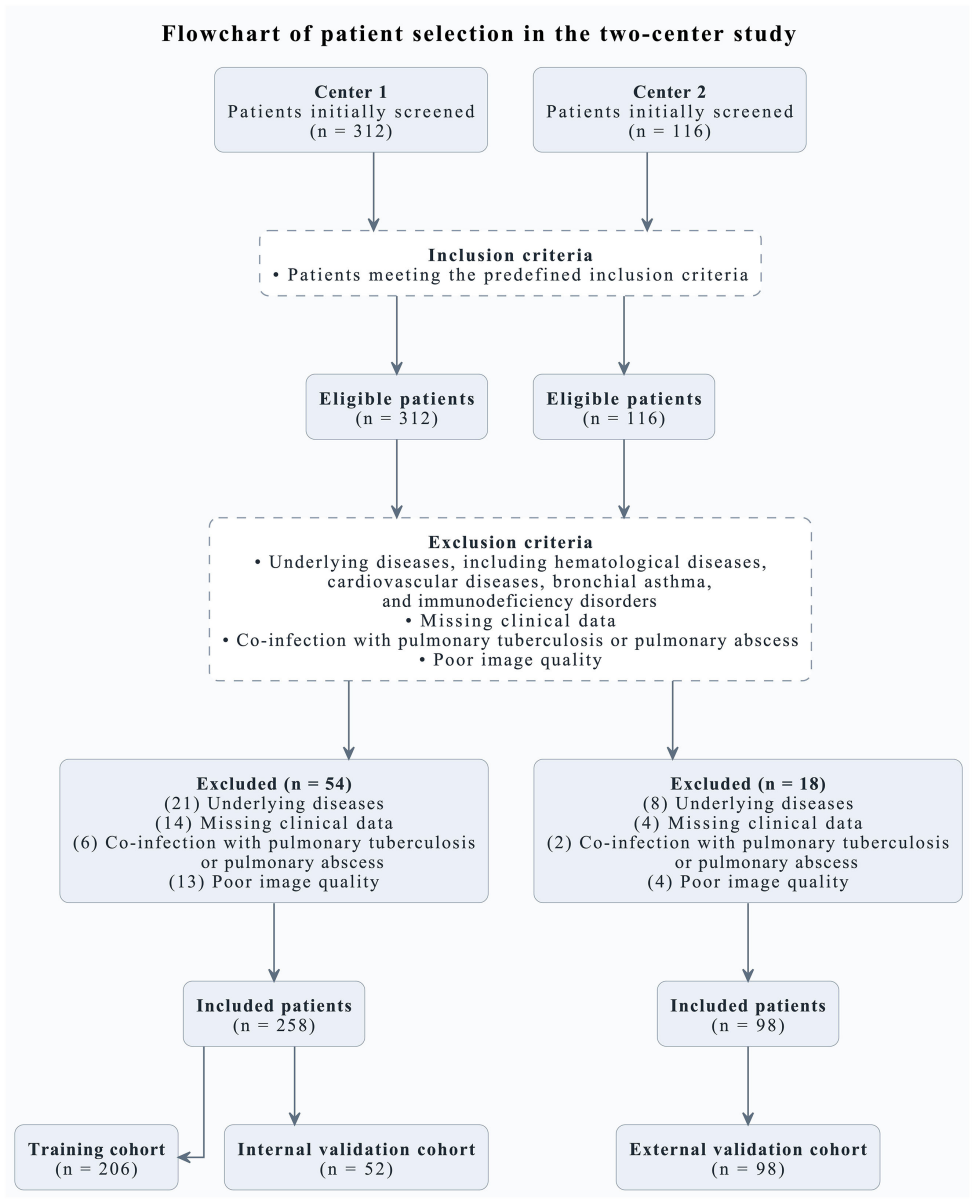
Flowchart of patient selection.

A total of 428 patients were initially screened for eligibility. After applying the inclusion and exclusion criteria, 356 patients were finally enrolled in this study. Specifically, 312 patients were screened in Center 1, of whom 54 were excluded for the following reasons: underlying diseases, including hematological diseases, cardiovascular diseases, bronchial asthma, and immunodeficiency disorders (n = 21); missing clinical data (n = 14); co-infection with pulmonary tuberculosis or pulmonary abscess (n = 6); and poor image quality (n = 13). The remaining 258 patients from Center 1 were included and subsequently allocated to the training cohort (n = 206) and the internal validation cohort (n = 52). In Center 2, 116 patients were initially screened, and 18 were excluded because of underlying diseases (n = 8), missing clinical data (n = 4), co-infection with pulmonary tuberculosis or pulmonary abscess (n = 2), and poor image quality (n = 4). Finally, 98 patients from Center 2 were included as the external validation cohort. The patient recruitment and cohort allocation process are shown in **Figure 2**.

**Table 1** summarizes the detailed clinical data of the patients, provide details of the descriptive statistics of continuous and categorical features of patients in the training/validation sets and test sets, respectively. Creatine kinase-MB (CK-MB), LDH, CRP, lobar atelectasis, bilateral lobe pneumonia, number of lobes involved, and lesion volume showed significant differences between the groups.

**Table 1:** General information of Clinical and CT feature with different cohort.

Characteristics	Training cohort			Validation cohort			Testing cohort			p-value
	Poor prognosiss(n=49)	Favorable prognosis(n=157)	p-value	Poor prognosis(n=12)	Favorable prognosis(n=40)	p-value	Poor prognosis(n=22)	Favorable prognosis(n=76)	p-value	
Gender (%)			0.182			0.022			0.053	0.660
Female	30 (61.224)	79 (50.318)		9 (75.000)	15 (37.500)		15 (68.182)	34 (44.737)		
Male	19 (38.776)	78 (49.682)		3 (25.000)	25 (62.500)		7 (31.818)	42 (55.263)		
WBC (Median[Q1,Q3])	8.000 [6.150,11.010]	7.860 [6.330,9.540]	0.684	9.215 [5.787,10.372]	7.600 [6.553,9.730]	0.508	10.545 [7.058,12.468]	8.575 [5.180,13.213]	0.199	0.310
EOS (Median[Q1,Q3])	0.100 [0.030,0.220]	0.090 [0.030,0.200]	0.800	0.080 [0.050,0.163]	0.065 [0.030,0.195]	0.579	0.045 [0.010,0.070]	0.060 [0.010,0.143]	0.262	0.001
BAS (Median[Q1,Q3])	0.000 [0.000,0.020]	0.010 [0.000,0.020]	0.403	0.010 [0.000,0.010]	0.010 [0.000,0.020]	0.471	0.025 [0.013,0.040]	0.020 [0.010,0.040]	0.876	<0.001
PLT (Median[Q1,Q3])	311.000 [222.000,378.000]	277.000 [227.000,347.000]	0.287	267.500 [228.500,294.500]	289.500 [244.500,375.500]	0.259	412.000 [242.750,492.500]	284.500 [233.000,365.750]	0.024	0.290
CK-MB (Median[Q1,Q3])	2.290 [1.800,3.150]	2.240 [1.800,2.680]	0.281	2.870 [2.453,3.160]	2.620 [1.875,2.900]	0.082	1.100 [0.625,1.425]	0.735 [0.500,1.300]	0.182	<0.001
LDH (Median[Q1,Q3])	320.000 [274.000,437.000]	276.000 [238.000,328.000]	0.000	261.500 [248.250,288.000]	306.500 [245.750,354.750]	0.042	272.000 [219.750,347.750]	255.500 [207.750,322.250]	0.211	0.008
APTT (Median[Q1,Q3])	33.900 [31.200,35.500]	33.400 [31.200,36.100]	0.789	32.850 [31.975,33.400]	32.800 [30.800,35.600]	0.853	31.500 [30.000,33.025]	32.100 [29.600,34.125]	0.247	<0.001
FIB (Median[Q1,Q3])	3.960 [3.370,4.420]	3.760 [3.370,4.230]	0.323	3.910 [3.775,3.973]	3.860 [3.400,4.232]	0.845	3.910 [3.333,4.740]	3.805 [2.985,4.715]	0.461	0.710
PCT (Median[Q1,Q3])	0.130 [0.070,0.230]	0.110 [0.070,0.170]	0.264	0.070 [0.070,0.095]	0.100 [0.060,0.193]	0.337	0.100 [0.100,0.190]	0.100 [0.100,0.180]	0.754	0.038
D-dimer (Median[Q1,Q3])	157.794 [98.124,337.246]	92.820 [64.090,155.142]	0.000	135.694 [109.505,186.635]	99.892 [70.831,186.635]	0.237	254.000 [174.250,388.000]	208.500 [137.000,333.500]	0.089	<0.001
CRP (Median[Q1,Q3])	12.880 [2.160,21.220]	8.150 [2.910,18.840]	0.478	5.825 [3.053,10.848]	5.730 [2.600,12.810]	0.769	4.575 [1.588,18.767]	5.850 [1.085,17.215]	0.818	0.077
Lobar_atelectasis (%)			0.027			0.264			0.645	<0.001
No	35 (71.429)	134 (85.350)		7 (58.333)	30 (75.000)		21 (95.455)	73 (96.053)		
Yes	14 (28.571)	23 (14.650)		5 (41.667)	10 (25.000)		1 (4.545)	2 (2.632)		
Consolidation_mixed_GGO (%)			0.832			0.450			0.878	<0.001
No	21 (42.857)	70 (44.586)		5 (41.667)	12 (30.000)		14 (63.636)	47 (61.842)		
Yes	28 (57.143)	87 (55.414)		7 (58.333)	28 (70.000)		8 (36.364)	29 (38.158)		
Halo_sign (%)			0.181			0.128			0.217	0.440
No	38 (77.551)	106 (67.516)		11 (91.667)	28 (70.000)		19 (86.364)	56 (73.684)		
Yes	11 (22.449)	51 (32.484)		1 (8.333)	12 (30.000)		3 (13.636)	20 (26.316)		
Cavitation (%)			0.575			NaN			NaN	>0.99
No	49 (100.000)	156 (99.363)		12 (100.000)	40 (100.000)		22 (100.000)	76 (100.000)		
Yes	0 (0.000)	1 (0.637)		0 (0.000)	0 (0.000)		0 (0.000)	0 (0.000)		
Nodule (%)			0.833			0.417			0.585	0.300
No	23 (46.939)	71 (45.223)		7 (58.333)	18 (45.000)		11 (50.000)	43 (56.579)		
Yes	26 (53.061)	86 (54.777)		5 (41.667)	22 (55.000)		11 (50.000)	33 (43.421)		
GGO (%)			0.473			0.188			0.307	0.660
No	19 (38.776)	70 (44.586)		8 (66.667)	18 (45.000)		8 (36.364)	37 (48.684)		
Yes	30 (61.224)	87 (55.414)		4 (33.333)	22 (55.000)		14 (63.636)	39 (51.316)		
Mediastinal_enlargement_of_lymph_nodes (%)			0.622			0.065			0.442	0.070
No	46 (93.878)	144 (91.720)		11 (91.667)	40 (100.000)		22 (100.000)	74 (97.368)		
Yes	3 (6.122)	13 (8.280)		1 (8.333)	0 (0.000)		0 (0.000)	2 (2.632)		
Air_bronchogram_sign (%)			0.790			0.239			0.447	<0.001
No	14 (28.571)	48 (30.573)		4 (33.333)	7 (17.500)		15 (68.182)	45 (59.211)		
Yes	35 (71.429)	109 (69.427)		8 (66.667)	33 (82.500)		7 (31.818)	31 (40.789)		
bronchial_wall_thickening (%)			0.806			0.361			0.094	0.070
No	34 (69.388)	106 (67.516)		10 (83.333)	28 (70.000)		15 (68.182)	64 (84.211)		
Yes	15 (30.612)	51 (32.484)		2 (16.667)	12 (30.000)		7 (31.818)	12 (15.789)		
Reticular_pattern (%)			0.402			0.357			0.857	0.070
No	43 (87.755)	144 (91.720)		11 (91.667)	39 (97.500)		20 (90.909)	70 (92.105)		
Yes	6 (12.245)	13 (8.280)		1 (8.333)	1 (2.500)		2 (9.091)	6 (7.895)		
Bilateral_lobe_pneumonia (%)			0.000			0.013			0.170	0.520
No	15 (30.612)	97 (61.783)		4 (33.333)	29 (72.500)		9 (40.909)	47 (61.842)		
Yes	34 (69.388)	60 (38.217)		8 (66.667)	11 (27.500)		13 (59.091)	28 (36.842)		
Number_of_lobes_involved (%)			0.000	2 (16.667)	16 (40.000)				0.025	0.400
1	11 (22.449)	63 (40.127)		6 (50.000)	6 (15.000)		5 (22.727)	42 (55.263)		
2	12 (24.490)	40 (25.478)		2 (16.667)	1 (2.500)		5 (22.727)	14 (18.421)		
3	5 (10.204)	33 (21.019)		0 (0.000)	5 (12.500)		5 (22.727)	4 (5.263)		
4	6 (12.245)	7 (4.459)					2 (9.091)	3 (3.947)		
5	15 (30.612)	14 (8.917)					4 (18.182)	8 (10.526)		
Lesion_Volume (Median[Q1,Q3])	114968.010 [70377.300,213767.760]	77234.060 [32011.920,145135.250]	0.001	107508.025 [74892.227,149336.242]	78199.285 [38541.683,112342.880]	0.254	62046.760 [26979.803,124406.788]	44932.320 [14331.932,134473.962]	0.461	0.033

WBC, White blood cell; PLT, platelet; CRP, C-reactive protein; PCT, procalcitonin; LDH, lactate dehydrogenase; APTT, activated partial thromboplastin time; BAS, Biotin-Avidin—System; EOS, eosinophil count; FIB, Fibrinogen.

**Figure 3:**
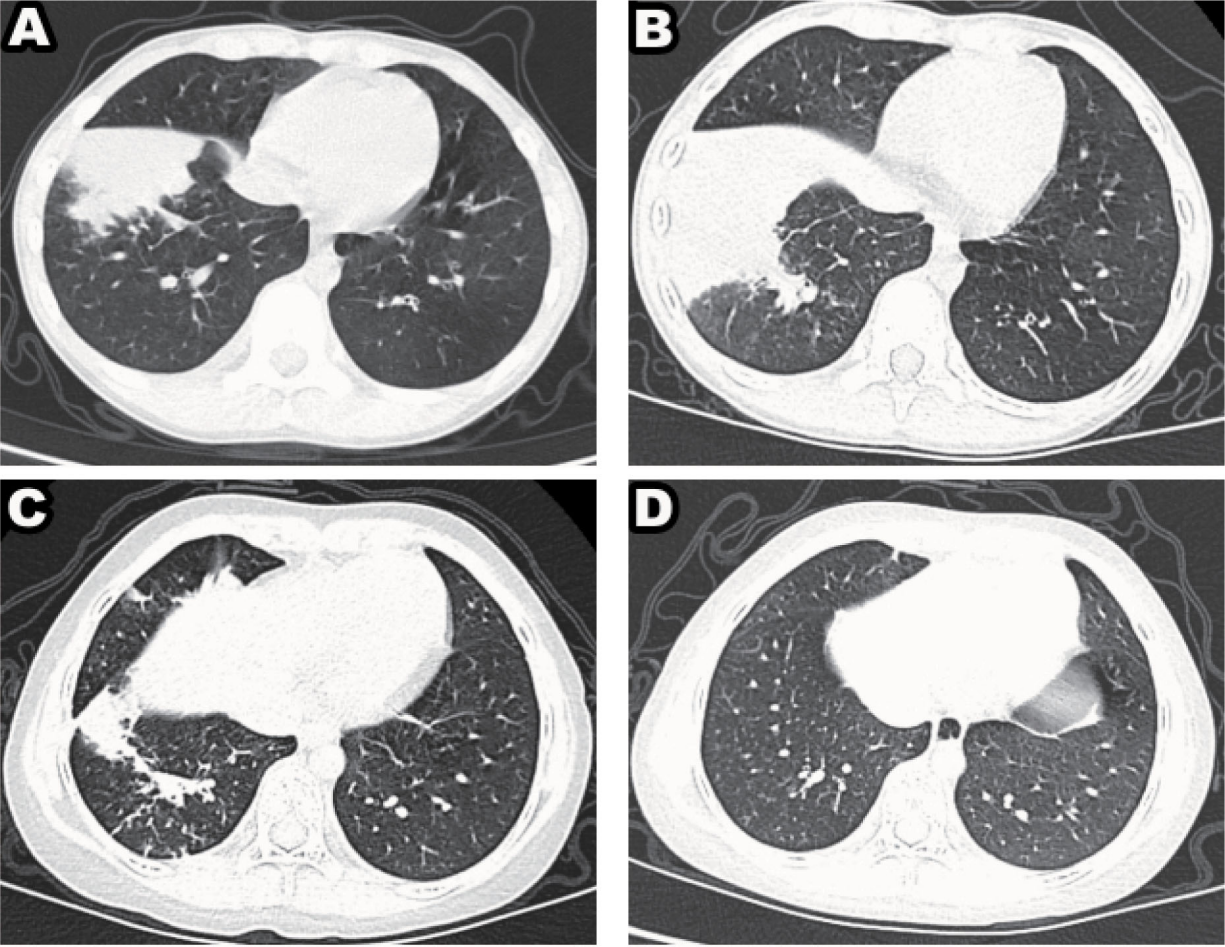
Mycoplasmal pneumonia in a child (male, 10 years old), mainly manifested as large patchy consolidation with poor prognosis, seven days later, CT image shows the extent of distribution increased **(A, B)**. Mycoplasmal pneumonia in a child (male, 12 years old) with favorable prognosis, seven days later, CT image shows the lesions to be absorbed **(C, D)**.

A univariate analysis was conducted for risk factors based on clinical and CT features. In the training set, the factors, namely CK-MB, LDH, CRP, lobar atelectasis, bilateral lobe pneumonia, and number of lobes, exhibited a significant prognostic value (all P < 0.05) and were selected to develop the clinical model. In the training, validation, and test cohorts, the clinical model based on the clinico-radiological risk factor alone was constructed. **Figure 3** shows the different prognosis of MP patients.

### 3.2 Segmentation reproducibility assessment

To evaluate the reliability and reproducibility of the semi-automatic lesion segmentation, the Dice similarity coefficient (DSC) was calculated on a randomly selected subset of 50 CT images. Intra-observer agreement was assessed by having the primary reader re-segment 50 cases after an interval of at least two weeks, while inter-observer agreement was evaluated by two independent radiologists (with 10 and 12 years of experience in thoracic imaging) segmenting the same subset independently. The mean intra-observer DSC was 0.92 (95% CI:0.85–0.97), and the mean inter-observer DSC was 0.89(95% CI: 0.82–0.95). These high DSC values indicate excellent intra- and inter-observer agreement, confirming the robustness of the segmentation process and supporting the reliability of subsequent radiomics feature extraction.

### 3.3 Radiomics features and prediction performance of the radiomics model

**Figure 4:**
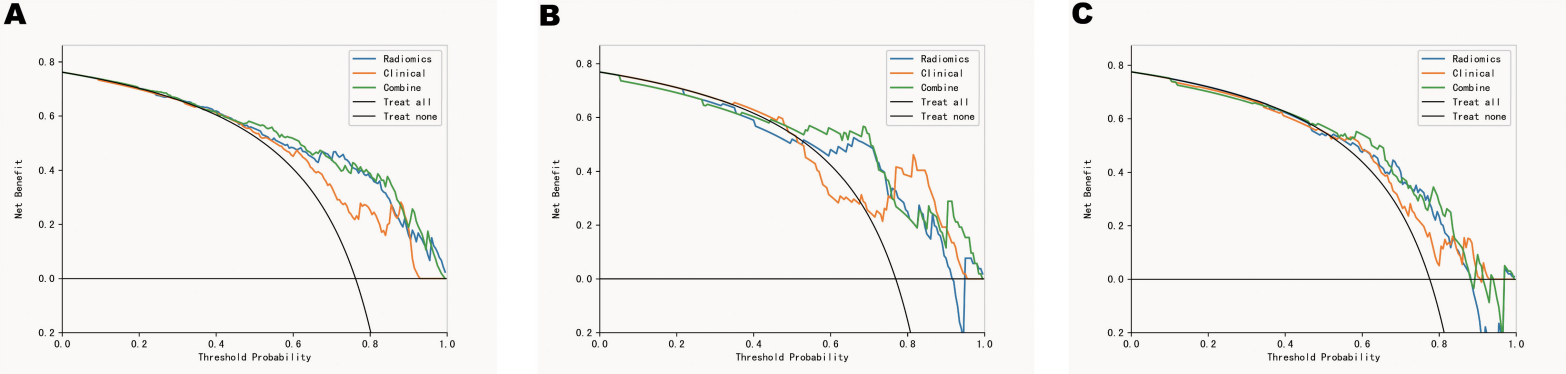
ROC curves in the training set **(A)**, validation set **(B)** and test set **(C)**.

**Figure 5:**
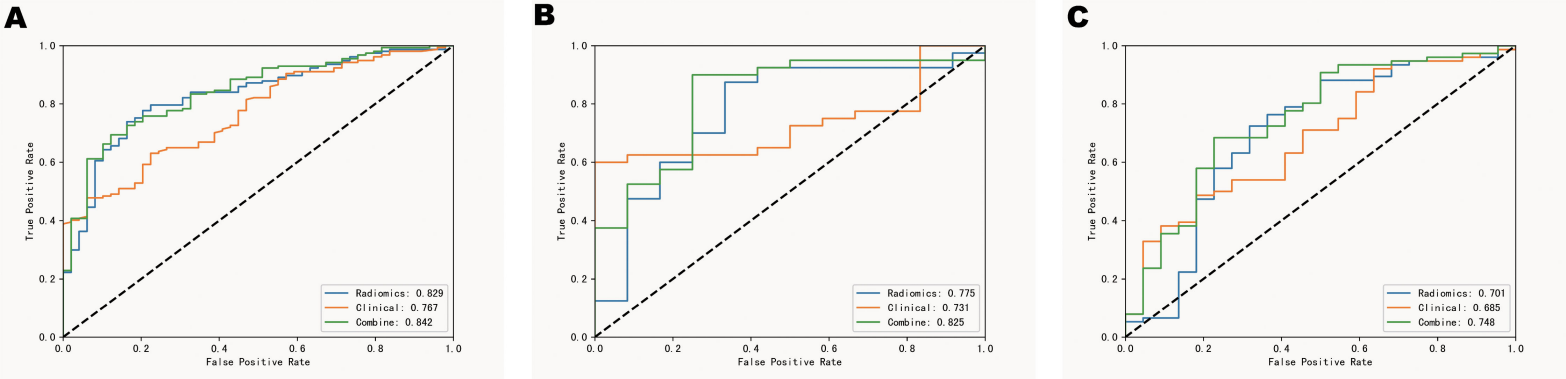
Decision curve analysis(DCA) for the radiomic model, clinical model and combine model in training set **(A)**, validation set **(B)** and test set **(C)**. The y-axis measures the net benefit.

A radiomics model was constructed based on the optimal radiomics features alone. This model showed AUC values of 0.829[95%CI: 0.773-0.880], 0.775[95%CI:0.617-0.897], and 0.701 [95% CI: 0.588-0.823] for identifying favorable and poor prognoses in the training, validation, and test cohorts, respectively. In the training cohort, the accuracy, sensitivity, and specificity of the model for evaluating favorable/poor prognosis were 0.762, 0.738, and 0.836, respectively. In the test and validation cohorts, the corresponding accuracy, sensitivity, and specificity were 0.769,0.653, 0.800 and 0.647,0.667,0.682, respectively. (**Table 2**; **Figure 4**). Decision curve analysis(DCA) for the radiomic model, clinical model and radiomics nomogram in training set, validation set and test set. (**Figure 5**).

**Table 2:** Predictive ability of four models for distinguishing different outcome of mycoplasmal pneumonia.

Model	Cohort	AUC(95% CI)	Sensitivity	Specificity	Accuracy
Clinical model	Training	0.767 (0.709-0.822)	0.739	0.837	0.762
	Validation	0.731 (0.632-0.850)	0.800	0.667	0.769
	Testing	0.685 (0.576-.0791)	0.644	0.681	0.653
Radiomics model	Training	0.829 (0.773-0.880)	0.738	0.836	0.762
	Validation	0.775 (0.617-0.897)	0.653	0.800	0.769
	Testing	0.701 (0.588-0.823)	0.667	0.682	0.647
Combine model	Training	0.842 (0.789-0.890)	0.694	0.877	0.737
	Validation	0.825 (0.700-0.928)	0.600	0.750	0.634
	Testing	0.748 (0.647-0.856)	0.592	0.772	0.632

### 3.4 Construction and validation of the clinical and radiomics model

We used backward stepwise competing risk regression with the minimum Akaike information criterion to select risk factors and construct a clinical and radiomics model. The clinical and radiomics model developed with Radscore and relevant clinical risk factors (CK-MB, LDH, CRP, bilateral lobe pneumonia, number of lobes, and lesion volume) showed significant improvement in predicting prognosis in the training cohort as compared to the clinical model (AUC = 0.842[95% CI: 0.789-0.890] vs. 0.767[95% CI:0.709-0.822]). Moreover, the clinical and radiomics model also showed significant improvement in predicting prognosis in the validation and test cohorts as compared to the clinical model (AUC = 0.825[95% CI: 0.700-0.928] vs. 0.731[95% CI: 0.632-0.850] and 0.748[95%CI:0.647-0.856] vs. 0.685[95%CI:0.576-.0791], respectively).

## 4 Discussion

The present study aimed to identify the relevance of radiomics features derived from CT images to predict the prognosis of children with MP. The results showed that the clinical and radiomics nomogram model that integrated imaging and clinical risk factors had a good predictive value for the prognoses of children with MP.

According to the study results, the LDH levels exhibited varying degrees of elevation between the favorable prognosis and poor prognosis groups. LDH is an essential enzyme in the glycolytic pathway and is widely present in human tissues and cells. LDH comprises five isoenzymes; the isoforms LDH1 and LDH2 are commonly found in the heart, kidneys, and red blood cells, while LDH3 shows higher levels in the lungs (Shin et al., 2014). Following M. pneumoniae infection, the body shows excessive immuno-inflammatory responses, leading to tissue cell damage due to the release of inflammatory factors. A previous study showed that the intracellular LDH concentration was approximately 1,500-fold higher than that in the serum (Yang et al., 2021). Moreover, the leakage of enzymes from extensively damaged tissues can significantly increase serum LDH concentration during acute hypoxia or inflammation. In addition to the damage to the respiratory system, MP can cause hypoxia and immune-related damage in organs/tissues such as the skin, gastrointestinal tract, blood, liver, and kidneys, leading to LDH release (Lu et al., 2011). An elevated serum LDH level can serve as a crucial laboratory predictor for assessing the prognosis of children with MP with pulmonary atelectasis. Thus, higher LDH levels indicate a more intense inflammatory response and a poor prognosis for children with MP. As shown in another study (Qiu et al., 2022), in severe pneumonia, the interaction between the inflammatory response and the clotting system can aggravate lung damage. Moreover, the incidence of pleural effusion and myocardial and liver damage was significantly increased in patients with severe MP with elevated D-dimer levels, resulting in increased CK-MB and LDH levels. This may reflect the mutual promoting effects of inflammation and clotting, thus aggravating systemic inflammation and leading to more severe symptoms and poor prognosis of patients.

Chest CT plays a crucial role in clinical diagnosis, monitoring disease progression, and evaluating treatment effectiveness (Sun et al., 2021). However, the role of CT in assessing the diagnosis and prognosis of MP remains controversial. Recent studies have shown that radiomics is a potential imaging approach that can identify the biological features of diseases and provide details more than those obtained through visual assessments based solely on CT images (Yang et al., 2023). A previous study used radiomics-based predictive models with random forest and k-Nearest Neighbor (kNN) classifiers to identify glucocorticoid-sensitive connective tissue disease-associated interstitial lung disease; the AUC values obtained for these models were 0.66 and 0.61, respectively (Feng et al., 2018). Chen et al (Chen et al., 2021). used three classifiers (kNN, support vector machine [SVM], and logistic regression [LR]) to estimate the prediction performance of the clinical factor model and the radiomics nomogram model for COVID-19 prognosis; the authors found that the AUC values of the radiomics models (kNN, SVM, and LR) were 0.88, 0.88, and 0.84, respectively, thus showing that the model exhibited good performance in predicting prognosis.

To the best of our knowledge, the present study is the first report on the application of chest CT-based radiomics for predicting prognosis in children with MP. The radiomics model exhibited better predictive performance (accuracy > 0.70 and AUC > 0.80) in the feature classification method. We conducted an in-depth analysis of the clinical factors and imaging findings of children with MP for their accurate diagnosis and treatment. The Radscore was then calculated, and a clinical and radiomics model was constructed based on independent clinical risk factors, radiomics features, and CT imaging findings; the model achieved good results in predicting the radiological progression of the lesions. The clinical and radiomics model showed higher AUC values and net benefits as compared to the clinical model; this finding suggested that CT-based radiomics features had an added value in differentiating favorable and poor outcomes in children with MP.

To address the interpretability of the selected 21 radiomics features and overcome the “black box” limitation, we provide a mechanistic interpretation linking these features to the underlying pathology of MP. The features were categorized into three main groups based on PyRadiomics definitions (1): First-order features (e.g., Kurtosis and Skewness), which quantify intensity distribution and peak sharpness; high Kurtosis often reflects heterogeneous tissue damage and necrosis due to intense immuno-inflammatory responses in MP, leading to uneven gray-level peaks from alveolar consolidation and interstitial edema (2). Shape-based features (e.g., Sphericity and Elongation), which describe lesion morphology; lower Sphericity indicates irregular, non-spherical shapes commonly seen in bronchopneumonia patterns with bronchial wall thickening and peripheral spread, while high Elongation may correspond to elongated consolidations along bronchovascular bundles (3). Texture features (e.g., GLCM_ClusterShade, GLCM_Homogeneity), which capture spatial heterogeneity; elevated ClusterShade (a measure of asymmetry in gray-level clusters) is associated with clustered inflammatory infiltrates and microvascular changes, potentially indicating more severe interstitial inflammation and poorer prognosis, whereas reduced Homogeneity reflects lesion irregularity from necrosis or patchy involvement (Aerts et al., 2014; Lambin et al., 2017).

During the development of the prognostic prediction models, we observed that the combined clinical and radiomics model showed the highest performance and remarkably outperformed the other models. Additionally, although the other models exhibited similar performance, significant differences were observed between them. The AUC values of the clinical model ranged from 0.685 to 0.767. In contrast, the AUC values of the clinical and radiomics model showed an increase and ranged from 0.748 to 0.842. These results suggested that the combined model provided more accurate information regarding the prognosis of children with MP.

The present study had some limitations. First, this was a retrospective cohort study; hence, potential selection bias could affect the reproducibility and stability of the results. The included patients had either more severe clinical symptoms or atypical symptoms as they were already hospitalized and had undergone CT scans. Second, Prognostic grouping in this study was primarily based on a composite assessment of clinical symptom improvement, radiological changes, and serological parameters; however, due to incomplete recording of key quantitative outcomes in the retrospective dataset, specific adverse event rates were not reported. And due to incomplete recording of key covariates in this retrospective study and the overall limited sample size, some subgroups were too small to yield robust performance estimates. Therefore, subgroup analyses according to age, disease severity, and antibiotic treatment were not performed in the present study. Future prospective multicenter studies with larger sample sizes and standardized data collection are warranted to support reliable subgroup validation.

## Data Availability

The original contributions presented in the study are included in the article/supplementary material. Further inquiries can be directed to the corresponding author.
